# A Trypsin Inhibitor from Tamarind Reduces Food Intake and Improves Inflammatory Status in Rats with Metabolic Syndrome Regardless of Weight Loss

**DOI:** 10.3390/nu8100544

**Published:** 2016-09-27

**Authors:** Fabiana M. C. Carvalho, Vanessa C. O. Lima, Izael S. Costa, Amanda F. Medeiros, Alexandre C. Serquiz, Maíra C. J. S. Lima, Raphael P. Serquiz, Bruna L. L. Maciel, Adriana F. Uchôa, Elizeu A. Santos, Ana H. A. Morais

**Affiliations:** 1 Postgraduate Nutrition Program, Center for Health Sciences, Federal University of Rio Grande do Norte, Natal RN 59078-970, Brazil; fabicoimbra@hotmail.com (F.M.C.C.); izaelsousa@hotmail.com (I.S.C.); brunalimamaciel@gmail.com (B.L.L.M.); 2 Postgraduate Biochemistry Program, Biosciences Center, Federal University of Rio Grande do Norte, Natal RN 59078-970, Brazil; vanessalima_nutri@yahoo.com.br (V.C.O.L.); amanda-nut@hotmail.com (A.F.M.); alexandreserquiz@gmail.com (A.C.S.); raphaserquiz@hotmail.com (R.P.S.); afuchoa2003@yahoo.com.br (A.F.U.); elizeu.ufrn@gmail.com (E.A.S.); 3 Course of Nutrition, Potiguar University, Natal RN 59056-000, Brazil; 4 Course of Veterinary Medicine, Potiguar University, Natal RN 59056-000, Brazil; mairalima4@hotmail.com; 5 Technical School Health, Potiguar University, Natal RN 59056-000, Brazil; 6 Department of Nutrition, Center for Health Sciences, Federal University of Rio Grande do Norte, Natal RN 59078-970, Brazil; 7 Tropical Medicine Institute (TMI), Federal University of Rio Grande do Norte, Natal RN 59078-970, Brazil; 8 Department of Cell Biology and Genetics, Center for Biosciences, Federal University of Rio Grande do Norte, Natal RN 59078-970, Brazil; 9 Department of Biochemistry, Center for Biosciences, Federal University of Rio Grande do Norte, Natal RN 59078-970, Brazil

**Keywords:** obesity, cafeteria diet, TNF*-*α, Glycemia

## Abstract

Trypsin inhibitors are studied in a variety of models for their anti-obesity and anti-inflammatory bioactive properties. Our group has previously demonstrated the satietogenic effect of tamarind seed trypsin inhibitors (TTI) in eutrophic mouse models and anti-inflammatory effects of other trypsin inhibitors. In this study, we evaluated TTI effect upon satiety, biochemical and inflammatory parameters in an experimental model of metabolic syndrome (MetS). Three groups of *n* = 5 male *Wistar* rats with obesity-based MetS received for 10 days one of the following: (1) Cafeteria diet; (2) Cafeteria diet + TTI (25 mg/kg); and (3) Standard diet. TTI reduced food intake in animals with MetS. Nevertheless, weight gain was not different between studied groups. Dyslipidemia parameters were not different with the use of TTI, only the group receiving standard diet showed lower very low density lipoprotein (VLDL) and triglycerides (TG) (Kruskal–Wallis, *p* < 0.05). Interleukin-6 (IL-6) production did not differ between groups. Interestingly, tumor necrosis factor-alpha (TNF-α) was lower in animals receiving TTI. Our results corroborate the satietogenic effect of TTI in a MetS model. Furthermore, we showed that TTI added to a cafeteria diet may decrease inflammation regardless of weight loss. This puts TTI as a candidate for studies to test its effectiveness as an adjuvant in MetS treatment.

## 1. Introduction

Metabolic syndrome (MetS) is an obesity-based condition characterized by the presence of insulin resistance (IR), high blood pressure and dyslipidemia. MetS clinical features include endothelial dysfunction and a pro-oxidant, pro-thrombotic, inflammatory condition [1,2].

The inflammatory process in MetS is due to the production of cytokines and chemokines by adipocytes. These inflammatory markers include TNF-α, IL-6, leptin, C-reactive protein (CRP), resistin, monocyte chemotactic protein (MCP-1), inducible nitric oxide synthase (iNOS), transforming growth factor-beta (TGF-β) and plasminogen activator inhibitor 1 (PAI-1). MetS commonly courses with adiponectin reduction, an anti-inflammatory adipokine [3].

Among molecular mechanisms associated with inflammation in obesity, is the signaling pathway of nuclear factor kappa B (NF-κB), which induces expression of several genes related to inflammation [4]. NF-κB pathway can be triggered by TNF-α binding to its receptor or by lipopolysaccharide (LPS) and saturated fatty acids binding to toll-like receptor 4 (TLR4) [4].

Thus, high ingestion of carbohydrates and saturated fatty acids or increased metabolic release of saturated fatty acids may activate inflammation, increasing risk for complications in obesity and MetS [4]. Monocyte CD14+ and TLR4 are also activated by TNF-α and interleukin-1α (IL-1α), commonly elevated in MetS [5,6,7]. Therefore, these cytokines are relevant molecular targets in the context of inflammation, MetS and nutrigenomics [5,6,7]. Furthermore, suppressing TNF-α production is a very important feature in the MetS treatment, once it acts as a potent mediator of the inflammatory process [8].

MetS treatment involves the use of several drugs, such as fibrates and thiazolidinediones (TZDs). These drugs act decreasing peripheral resistance by increasing insulin sensibility in the liver, muscles and adipocytes [9]. In addition, they activate nuclear peroxisome proliferator activated receptor γ2 (PPARγ2), which regulates expression of genes related to lipid and glucose metabolism, glucose uptake mediated by insulin in peripheral tissues and differentiation of preadipocytes into adipocytes [10].

TZDs increase the expression of glucose transporters 4 (GLUT4) in muscles and adipocytes, the action of lipoprotein lipase (LPL) and reduce TNF-α and leptin expression [11,12]. Taken together, these actions decrease inflammation in MetS. Nevertheless, side effects, especially hepatotoxicity, caused by fibrates and TZDs have led to an increase in research focusing on less toxic substances, with more selective properties. Hence, plants represent a source of bioactive substances, and their use in studies aiming the formulation of new biopharmaceuticals has been greatly intensified. 

Protease inhibitors, especially those directed to trypsin, are studied as biopharmaceutical drug candidates. Their properties vary among anti-coagulant, anti-inflammatory, anti-cancer, anti-bacterial, anti-fungal and anti-obesity [8,13,14,15]. Studies have also demonstrated that trypsin inhibitors can inhibit human neutrophil elastase (HNE) and reduce inflammatory cytokines [16,17,18,19]. Furthermore, trypsin inhibitors increase cholecystokinin (CCK), a satietogenic hormone, which may be the link to reduced food intake and weight loss seen in studies using seeds rich in trypsin inhibitors [15,18,20,21,22]. These studies found that trypsin inhibitors act as CCK inducers, confirming the peripheral and central action of these inhibitors in satiety [23,24,25]. Furthermore, studies have demonstrated that CCK synergizes and has a functional effect with other gastrointestinal hormones secretion, such as leptin and ghrelin, which are also satietogenic [26,27]. This possibly promotes the effect of CCK.

Some studies have purified and characterized trypsin inhibitors in Tamarindo seeds [17,28]. Other recent study conducted by our group [20] isolated a trypsin inhibitor from Tamarindo seeds (Tamarindo seed Trypsin Inhibitor (TTI)), which presented a satietogenic effect, reducing food intake and weight gain and increasing CCK production in eutrophic rats. Nevertheless, it is well known that in lean individuals inflammatory adipokines are much lower when compared to obese [11], which was a limitation of our previous experimental approach to test TTI anti-inflammatory effect. 

Researchers commonly use experimental models to understand the role of each element involved in the pathophysiology of MetS. These models can better control the role of each of the components of insulin resistance and obesity. Besides, experimental models represent first in vivo experiments for new therapy testing [29]. 

Studies have clearly debated that MetS characterization in animals is not well established. However, MetS is induced using cafeteria diets, which reflect the variety of energy-dense and palatable foods (usually industrialized products), prevalently consumed in the Western society, and associated with obesity, hypertension, hyperinsulinemia, insulin resistance, hyperglycemia and hypertriglyceridemia [29,30,31,32,33,34].

Carbohydrate rich diets commonly cause an increase in adipose tissue by an increase in PPARγ2 expression, which regulates adipogenesis [30]. In adipocytes, PPARγ2 regulates the expression of several genes involved in lipid metabolism, including Adipocyte protein 2 (aP2) [31], acil-CoA sintetase [32] and LPL [33]. It also controls the expression of the fatty acid transporter protein 1 (FATP-1) and the cluster of differentiation 36 (CD36) [34], both involved in lipids uptake by adipocytes. 

The effect of trypsin inhibitors increasing satiety and reducing inflammation in lean models [8,15,17,18,19,20,21,22], led us to hypothesize that these actions can be seen in an obesity-based MetS model, where high food intake and inflammation play hallmark roles. Thus, we obtained TTI and tested it in *Wistar* rats with obesity-based MetS, assessing its effect in satiety, weight gain and inflammation. Results have the potential to stimulate studies for new drugs and adjuvants for MetS prevention and treatment.

## 2. Materials and Methods

### 2.1. Extraction, Fractioning and Isolation of the Trypsin Inhibitor

We isolated a trypsin inhibitor form tamarindo seeds (TTI), an endemic fruit in northeast Brazil, using steps previously determined by Ribeiro et al. [20]. Tamarindo fruit was locally purchased in the city of Natal, Rio Grande do Norte, Brazil. Seeds were removed from pulp and peeled off using a stylet for obtaining cotyledons. 

The peeled seeds were mashed at 6 °C until a flour of 40 mesh. Then, 50 mM Tris-HCl buffer, pH 7.5 at 1:10 (*w*/*v*) was added for extraction. The solution was submitted to constant agitation for 3 h at room temperature, centrifuged (10,000× *g* for 30 min at 4 °C) and filtered to obtain the crude extract (CE) [20].

Protein fractioning was done by sequential precipitation with ammonium sulfate in saturation ranges of 0%–30%, 30%–60% and 60%–90% in agitation under room temperature, following centrifugation (10,000× *g* for 30 min at 4 °C). Precipitated fractions were suspended in 50 mM Tris-HCl buffer, pH 7.5 and dialyzed against the same butter. After dialysis, the fractions were referred to as F1 (0%–30%), F2 (30%–60%) and F3 (60%–90%) and stored at −20 °C. All fractions were analyzed for their trypsin inhibition activities [34] using 1.25 mM BApNA (Nbenzoyl-dl-arginine-*p*-nitroanilide) as a substrate. 

Once F2 presented the highest trypsin inhibition activity, it was submitted to a Trypsin-sepharose affinity chromatography (10 cm × 1.5 cm) in 50 mM Tris-HCl buffer, pH 7.5 for TTI isolation. Adsorbed proteins were eluted from the matrix with 5mM HCl solution and collected in aliquots of 3 mL at a flow rate of 0.5 mL/min. The protein profile was evaluated by spectrophotometry at 280 nm (Ultrospec™ 2100 pro UV/Visible spectrophotometer, GE Healthcare Bio-Sciences Corp., Piscataway, NJ, USA). Adsorbed proteins in the affinity column were dialyzed against 50 mM Tris-HCl buffer, pH 7.5, lyophilized and subjected to trypsin inhibition assay [35] to confirm trypsin inhibition action and referred to as TTI.

Protein quantification was done using Bradford methodology [36] using bovine serum albumin (BSA) as a standard. The results of the trypsin inhibitor specific activity were expressed as residual activity of the enzyme calculated from the hydrolysis promoted in the absence of inhibitor (100% of enzyme activity). Specific activity was expressed in IU/µg of soluble proteins, where one inhibition unit (IU) is the amount of inhibitor that reduces 0.01 in the absorbance at 410 nm (Ultrospec™ 2100 pro UV/Visible spectrophotometer, GE Healthcare Bio-Sciences Corp., Piscataway, NJ, USA) in the activity assay.

The material was frozen and stored at −20°C. Isolation and estimated molecular weight of TTI were evaluated by discontinuous and denaturing 12.5% sodium dodecyl sulfate polyacrylamide gel electrophoresis (SDS-PAGE) [37]. An aliquot containing approximately 30, 15 and 10 µg (CE, F2 and TTI, respectively) was applied on the gel. After electrophoresis, proteins were silver stained [38].

All in vitro assays occurred in the Laboratory of Chemistry and Function of Bioactive Proteins, at Federal University of Rio Grande do Norte State, Brazil. All of the reagents used were of analytical grade and obtained from Sigma (St. Louis, MO, USA) or VETEC Fine Chemicals Ltd., (Rio de Janeiro, Brazil).

### 2.2. In Vivo Experiment

#### 2.2.1. Animals and Experiment Design

Male, adult, obese *Wistar* rats (350–450 g) with MetS were provided by Potiguar University vivarium. This obesity was previously induced using a cafeteria diet (Table 1) in rats with 4 weeks of age for 17 weeks. This same cafeteria diet was used during our in vivo experiment. Animal were kept under standard conditions of light (12/12 h light/dark cycle) and temperature (23–25 °C) with water and food ad libitum. All experiments were performed in accordance with the Guide for the Care and Use of Laboratory Animals [39] and the study was approved by the Ethics Committee on Animal Use (EUA-UNP) under No. 012/2015. After confirmation of obesity and MetS, animals were divided in three groups, submitted to an adaptation period of five days, followed by 10 days of one of these diets:

- Cafeteria diet (*n* = 5): cafeteria diet + 1 mL of gavage water. This group was considered the control group, which did not receive treatment.

- Cafeteria diet + TTI (*n* = 5): cafeteria diet + 1 mL of gavage TTI (25 mg/Kg). This was considered the test group and the same dose given by Ribeiro et al. [20] was used.

- Standard diet (*n* = 5): Labina^®^ diet + 1 mL of gavage water. This group was considered the group receiving conventional treatment.

On Day 11, rats were sacrificed to collect blood for biochemical and inflammatory parameters.

#### 2.2.2. Diets

Standard diet given to rats was Labina^®^ while the cafeteria diet was produced in our Laboratory. This last one used Labina^®^ added to high glycemic index foods, as proposed by Naderali et al. [40] (Table 1). This same cafeteria diet was also used to induce obesity and MetS in the studied animals.

#### 2.2.3. Obesity and Metabolic Syndrome (MetS) Confirmation

Animals had obesity and MetS confirmed following Novelli et al. [41] recommendations and considering variables used in humans as proposed by the National Cholesterol Education Program Adult Treatment Panel III—NCEP ATP III [42]. Thus, altered Lee index, waist circumference, fasting glucose, high-density lipoprotein (HDL-C) and triglycerides (TG) were considered for diagnosis.

The same researcher measured naso-anal length, thoracic and waist circumference. Lee index was calculated and classified as proposed by Novelli et al. [41].

Animal blood was collected by cardiac puncture 72 h before adaptation. For that, they were fasted for 8 h and anesthetized with 250 mg of Tiletamine (cloridrate) and 250 mg of Zolazepam (cloridrate). The following biochemical parameters were analyzed: fasting plasma glucose, TG and HDL-C once these are used for the diagnosis of MetS in humans [42] and cited as the standard for diagnosis in experimental models [41]. The method used for the measurements of biochemical parameters was the enzymatic colorimetric (CELM^®^ Kit, São Paulo, Brazil).

As normal reference values, we used average data of five male, adult, eutrophic (320–380 g) *Wistar* rats, acclimatized under the same conditions, consuming Labina^®^ standard diet, as shown in Table 2. All values used were in accordance to Guimarães [43].

#### 2.2.4. Food Intake and Weight Gain

Rats were randomly and individually assigned in polypropylene cages in three groups. The five days of adaptation were used to establish the pattern of food consumption of each animal. During this adjustment period, all animals received water by gavage and all procedures from the 10 days of experiment were daily performed. During the experiment, food intake and weight were evaluated 1 h after diet administration. Animals were fasted 6 h before oral administration of diets. The results of daily food intake and weight were expressed using the differences day by day throughout the experiment and as percentages (%), using the initial and end values of the experiment. 

#### 2.2.5. Biochemical and Inflammatory Parameters

At the end of the experiment (day 11), animals were fasted for 8–12 h before the diet was given, and after 1 h from gavage, blood was collected by portal vein and used for glucose, insulin, TG, HDL-C, low-density lipoprotein (LDL-C), very low-density lipoprotein (VLDL-C), total cholesterol, glutamic oxaloacetic transaminase (SGOT), glutamic pyruvic transaminase (SGPT), gamma-glutamyl transpeptidase (GGT), TNF-α and IL-6 determination. After this, animals were euthanized in a CO_2_ chamber. The method used for biochemical parameters measurement was the enzymatic colorimetric (Kit CELM^®^, São Paulo, Brazil).

Serum samples were analyzed using commercially available immunoassay kits, according to Vendrame et al. [44]. Quantikine Rat TNF-α Immunoassay (R&D Systems # RTA00, São Paulo, Brazil) and Quantikine Rat IL-6 Immunoassay (R&D Systems # R6000B, São Paulo, Brazil) were used. According to manufacturer, kit sensitivities were <15 pg/mL and <30 pg/mL for TNF-α and IL-6, respectively.

### 2.3. Statistical Analysis

Data was analyzed for normality using the Kolmogorov-Smirnov test. All variables were considered nonparametric, and Kruskal–Wallis test was used to evaluate if daily differences in weight gain and food intake, biochemical and inflammatory data differed between studied groups. When significant differences were found, Dunn’s post-hoc test was used. From daily differences in weight gain and food intake, box plots were constructed for comparison between study groups. These graphs, show median values and were important for the visualization of the dispersion/homogeneity in the different groups. To test whether there was a correlation between weight gain and food intake, Spearman correlation was used (*r*^2^). Data was expressed as means ± SD and analyzed using Graph Pad Prism, version 5.0 (Graph Pad Software, San Diego, CA, USA). Significant differences were accepted when the *p* value was less than 0.05.

## 3. Results

### 3.1. TTI Isolation in Trypsin-Sepharose Affinity Chromatography

**Figure 1:**
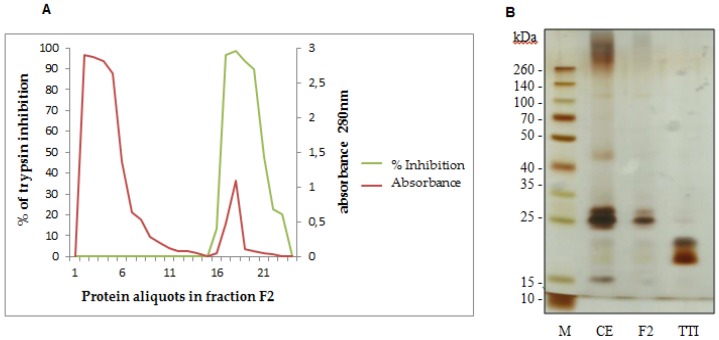
(**A**) Trypsin-sepharose affinity chromatography profile of fraction F2 obtained from Tamarindo seeds. The first peak (red line) represents proteins with low trypsin affinity, firstly eluted, with no trypsin inhibition activity (green line). The second peak (red line) represents TTI, with high trypsin affinity and trypsin inhibition activity (green line). The trypsin-sepharose affinity column was calibrated with 50 mM Tris-HCl, pH 7.5 buffer. Eluition was done using a 5 mM HCl solution, at a flow rate of 0.5 mL/min. The percent of trypsin inhibition of the eluted proteins was evaluated using a trypsin inhibition assay [35] with 50 µL of protein aliquots. Protein aliquots (3.0 mL) were observed at 280 nm. (**B**) Proteins were studied with 12.5% SDS-Polyacrylamide Gel Electrophoresis stained with silver nitrate of Tamarindo seeds. M: Marker (São Paulo, Brazil) (Spectra Multicolor Broad Range Protein Ladder); CE: Crude Extract; F2: fraction F2 saturated with 30%–60% of ammonium sulfate; TTI: Tamarindo seed Trypsin Inhibitor.

**Figure 2:**
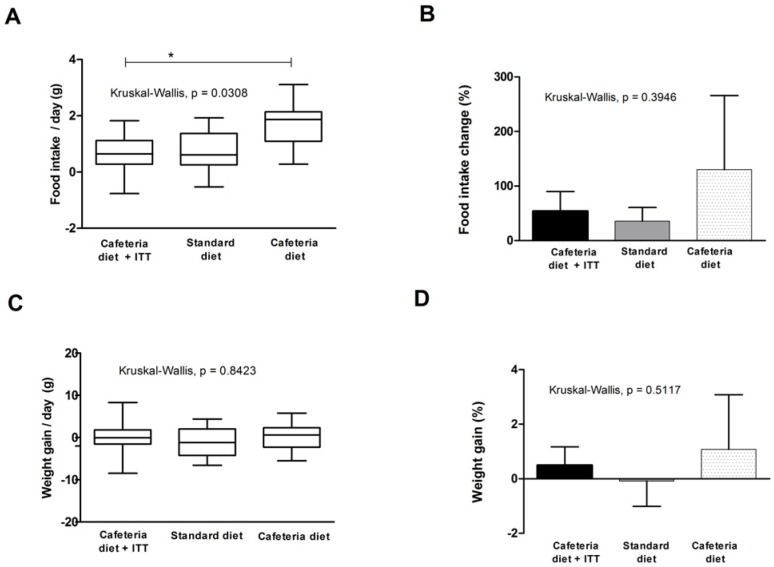
Food intake and weight gain in *Wistar* rats with obesity-based MetS submitted to different treatments: Cafeteria diet + TTI (cafeteria diet + 1 mL of gavage TTI at 25 mg/Kg); Standard diet (Labina^®^ diet + 1 mL of gavage water); Cafeteria diet (cafeteria diet + 1 mL of gavage water). All groups represent experiments with *n* = 5 animals. (**A**) Daily differences in food intake (g) box plots of study groups; (**B**) total food intake change (%) in studied groups; (**C**) daily weight gain (g) box plots of study groups; and (**D**) total weight gain (%) in studied groups. * Statistical difference between groups, with Dunn’s post-hoc test *p* < 0.05. TTI: Tamarindo seed Trypsin Inhibitor.

We obtained TTI using a trypsin-sepharose affinity chromatography of fraction F2, which presented the highest inhibitory activity against trypsin (Figure 1A). Fraction F2 was applied in the trypsin-sepharose column, and eluted using HCl at 5 mM. Figure 1A represents the inhibition percent (green line) and absorbance (red line) of the eluted peaks. The first red peak represents proteins in fraction F2 that were not adsorbed in the column and do not inhibit trypsin (green line). The second red peak represents TTI. TTI presented 83,970.58 UI/mg of protein and 100% trypsin inhibition. TTI isolation was confirmed by 12.5% SDS-PAGE, demonstrating the presence of two protein bands, with the predominance of the molecular mass of approximately 20 kDa (Figure 2B).

### 3.2. Metabolic Syndrome (MetS) Confirmation

As shown in Table 3, all studied animals were obese and presented MetS, according to the Lee index, waist circumference and fasting glucose. Additionally, almost all animals presented at least one more altered parameter, either TG or HDL-C when compared to the normal values listed in Table 2.

### 3.3. Food Intake and Weight Gain

TTI was tested to evaluate its effects in food intake and weight gain in animals with obesity and MetS for 10 days and these results are shown in Figure 2. In Figure 2A, data represent median daily differences in food intake (g), considering 10 days of experiment. Kruskal–Wallis test indicated a significant difference between studied groups (*p* = 0.0308), where the group treated with TTI presented the lower medians, significantly different from the cafeteria diet group (Dunn’s post-hoc test, *p* < 0.05), but similar to the standard group. This figure also shows the scatter/homogeneity of the data, demonstrating that the group treated with TTI showed less scattered and more homogenous results. 

Figure 2B shows the difference in food intake, considering final and initial mean values, in percentage (%), demonstrating that the cafeteria group had the highest increase in food intake, followed by the TTI treated group and the standard diet group. 

Figure 2C shows median weight gain (g) per group during the 10 day of study period. No statistical difference was seen between the studied groups (Kruskal–Wallis test, *p* = 0.8423). Nevertheless, in Figure 2D, in which total weight gain is shown, considering final and initial mean values, in percentage (%), it is possible to observe that the cafeteria group showed the highest weight gain, although not statically different between groups. 

We performed Spearman analysis to verify if food intake and weight gain correlated in this study. No significant correlations were found in the TTI treated group (*r*^2^ = −0.4061, *p* = 0.2475), standard diet group (*r*^2^ = 0.6242, *p* = 0.0603) or cafeteria group (*r*^2^ = 0.1515, *p* = 0.6821).

### 3.4. Biochemical and Inflammatory Parameters

To test if TTI had an effect in the metabolic and inflammatory process of MetS, biochemical parameters and cytokines were measured following 10 days of experiment, as shown in Table 4. For biochemical variables, groups were not different after treatment, except for the standard diet group, which presented the lower TG (62.2 ± 18.0 mg/dL) and VLDL-C (12.4 ± 3.6 mg/dL) concentrations, when compared to the other groups (Kruskal–Wallis *p* = 0.0108).

Nevertheless, a tendency to lower fasting glucose was observed in the cafeteria + TTI and standard diet group when compared to the cafeteria diet group. In percentages, animals treated with TTI and with standard diet presented values of fasting glucose 44% and 59% lower than those presented by animals receiving only the cafeteria diet, respectively. Transaminases (SGOT, SGPT and GGT) were also not different between studied groups. 

Concerning inflammatory cytokines (Table 4), IL-6 did not differ between studied groups. Interestingly, for TNF*-*α, all animals treated with TTI presented undetectable concentrations, which was not observed in the other studied groups. 

## 4. Discussion

The current increase in overweight/obesity and the growing number of MetS cases is a worldwide public health problem [2]. This made the evaluation of new molecules with potential to promote satiety and modulate metabolic changes in the inflammatory context of MetS an important research field.

In this sense, although classically known by their antinutritional digestive effects, trypsin inhibitors have been studied as satiety inducers [15,18,20,21,22]. In a previous study [20], our group isolated TTI and observed overall food intake decrease associated with increased serum CCK and reduced weight gain in a eutrophic experimental model. This was an interesting result once no protein digestion reduction was observed [20].

Hence, in the present study, we isolated TTI and tested its effect in an obesity-based MetS model. Our experimental obesity and MetS were confirmed using previously described parameters [41,42]. By comparing data from Table 1 and Table 2, alteration in all analyzed parameters is shown. These altered conditions in the study animals were induced using a cafeteria diet, developed and produced by our group.

Several studies show cafeteria diets commonly cause hypertension, hyperinsulinemia, hypertriglyceridemia, and insulin resistance [29,45,46,47,48]. Changes in serum glucose, body weight, adipose tissue and HDL-C seem to be less common [29,48]. Nevertheless, the cafeteria diet used for MetS induction was efficient to change parameters not commonly altered, such as fasting glucose and body weight, possibly because our diet might have caused insulin resistance. 

In other studies, using experimental obesity models, this condition has also been favored with the use of nutritionally inadequate diets that resemble feeding habits of the modern society. Nascimento et al. [28] developed a cycle of high fat diet to promote obesity in rats, and used as diagnostic parameters body weight, biochemical and hormonal data, systolic blood pressure and oral glucose tolerance test. Marques et al. [48], using a high fat diet in adult *Wistar* rats, promoted obesity and evaluated TG, total cholesterol, HDL-C, VLDL-C, glucose, weight, waist circumference and the Lee index. 

Here we demonstrate, for the first time, the satietogenic effect of TTI in an obesity based-MetS model. Animals treated with TTI presented lower food intake when compared to the other studied groups. This could be possibly explained by CCK increase, once this was observed in other studies [15,18,20,21,22]. CCK effect in satiety can be also explained by its synergic effect with other hormones, increasing leptin and inhibiting ghrelin secretion, which are also responsible for appetite control [26,27].

It is important to highlight that animals treated with TTI continued the use of the cafeteria diet, and only one group was submitted to a standard nutritionally balanced diet. Even so, we observed an effect of TTI on satiety, and TTI treated animals had a food intake very similar to those consuming a standard diet. This last group naturally reduced food intake by consuming a nutritionally balanced diet. 

Considering weight gain (%), a similar pattern to food intake is observed, but there were no statistically significant differences between groups. However, there was increased weight gain (%) in the cafeteria diet group. In addition, there was a considerable reduction in weight gain (%) in the TTI group and standard diet.

McLaughlin et al. [49] examined the effect of the synthetic trypsin inhibitor *N*,*N*-dimethyl-4-(4-guanidino-benzyloxy)-phenyl methyl sulphate (DGPM) in models of obese and eutrophic mice. DGPM administration (25 to 200 mg/kg) in rats after 6 h of fasting decreased food intake dose-dependently. A decrease in the average size of meals was observed in both obese and eutrophic rats. Taken Ribeiro et al. [20] and our results together, we can state that TTI has the ability to reduce food intake in both eutrophic and obese models, respectively. 

However, TTI seems to have a better effect on reducing food intake and consequently weight gain in eutrophic animals. This may be related to the fact that, in the study conducted by Ribeiro et al. [20], a standard diet was used to test TTI effect. In the present study, besides being obese, our animals continued consuming a cafeteria diet when treated with TTI. Thus, we believe that TTI effect, in obese animals with MetS could be increased if administered together with a nutritionally adequate diet.

Other studies have corroborated the reduction of food intake by trypsin inhibitors, attributing this effect to CCK induction [15,18,20,21,22]. Komarnytsky et al. [22] reported that oral administration of a concentrated potato trypsin inhibitor (CIPP) was efficient in reducing food intake and weight gain in eutrophic rats. This was due to increased CCK by a trypsin-dependent mechanism. 

A potato extract (Potein) with 60% of carbohydrates and 20% of proteins, including trypsin inhibitors, was tested in eutrophic rats. Three different concentrations of Potein (100 mg/kg, 200 mg/kg and 400 mg/kg) reduced food intake through CCK secretion. The proposed mechanism of action was a direct effect of Potein in enteroendocrine cells, with reduction in food intake between 1 and 3 h after Potein administration [21]. 

Serquiz et al. [15] showed that a protein isolate from peanut with anti-trypsin activity (AHTI) was able to reduce food intake and weight gain in a healthy experimental model. This was also attributed to the increased CCK concentration in rats receiving AHTI. 

Currently, protein inhibitors are used as commercial natural nutraceutical drugs to reduce food intake, proving their potential to application in humans. Potein^®^ (Hokkaido University, Sapporo, Japan), as a registered product (number 79075065), is an example [18]. In addition, pure molecules with inhibitory actions have been synthesized and used as drugs for various treatments. As example, are drugs that reduce digestion and absorption of nutrients, such as orlistat, which binds, in the intestinal lumen, to lipoprotein pancreatic lipase, reducing its action and thus TG digestion [50]. In addition, specific serotonin reuptake inhibitors have been used in the treatment of obesity. As an example, fluoxetine is indicated when obesity is associated with depression, anxiety or binge eating. Sibutramine is a reuptake inhibitor of serotonin, norepinephrine and dopamine used in the treatment of obesity [51]. These drugs demonstrate that pure molecules might have a better effect on satiety. Thus, future steps of our research will address on purifying protein from TTI, once we hypothesize that this might potentiate the results here demonstrated. 

Studies have also shown that enzymatic inhibitors act regulating diverse biochemical parameters. Serquiz et al. [15] observed AHTI effect reducing fasting glucose in healthy animals. In the present study, we did not observe statistical differences between groups concerning total cholesterol, LDL-C and HDL-C. Only VLD-C and TG were significantly lower in the group treated with the standard diet. This may be explained by the fact that the standard diet was adequate in carbohydrates and lipids, whereas the cafeteria diet was rich in carbohydrates. 

Nevertheless, a tendency for lower fasting glucose in TTI and standard groups was observed. This may be explained by the reduction in food intake in these groups, which might have decreased the effect of the rich carbohydrate diet in the group consuming the cafeteria diet with TTI. Possibly, the metabolic changes of each animal, generating high standard deviations, commonly seen in experimental models [52,53], might explain the results with no statistically significant differences.

The effect of a satietogenic trypsin inhibitor on decreasing fasting glucose, was shown in a study conducted by our group [15]. However, other studies using trypsin inhibitors also did not observe an effect on biochemical parameters [18,21,22,49,54]. Ribeiro et al. [20] did not observe any effect of TTI on the same biochemical parameters shown in the present study. This indicates that TTI may have differentiated actions other than changing biochemical variables. 

In this study, transaminases (SGOT, SGPT and GGT) were analyzed and their values were not different between groups. This corroborates the findings of Ribeiro et al. [20], who also analyzed the same enzymes, stomach, intestine, pancreas and liver histopathology and found no alterations in TTI (25 mg/kg) treated animals. Together, these data suggest that TTI is harmless to the hepatic tissue, as proposed by Ribeiro et al. [20]. These results in both studies are an indicator that TTI might be a safe oral biopharmaceutical candidate. 

Besides characteristic biochemical alterations, MetS courses with inflammation directly associated to the increased adipose tissue [3,52,55]. Here we analyzed IL-6 and TNF-α as inflammation markers. IL-6 was not changed with any of the tested treatments and TNF-α was reduced to undetectable concentrations in all animals treated with TTI. 

This is an interesting result once TNF-α plays central role in MetS inflammation [5,6,7]. Furthermore, considering that TNF-α concentrations are undetectable in healthy humans and animals [56,57], we can state that TTI presents an important effect in reducing inflammation in obese-based MetS animals to levels of healthy animals. 

TNF-α is a cytokine with paracrine and endocrine actions, produced by adipocytes, macrophages, skeletal and smooth muscle cells and endothelial cells. It is well known that this cytokine modulates other cytokine expression and is involved in low chronic inflammation processes, such as the ones seen in obesity and MetS [55]. 

In the present study, all animals were obese and presented MetS. Strikingly, TTI treatment reduced inflammation even though no alterations in weight gain were observed. This is an interesting effect once TTI was also able to reduce food intake from a cafeteria diet. Taken altogether, our results are promising, once translating an experimental standard diet to a clinical balanced diet for obese humans with MetS is difficult, as obese individuals may be reluctant in changing food habits. Hence, proven effective, TTI might act as a therapy adjuvant, helping to reduce not only food intake from an inadequate diet, but also inflammation regardless of weight loss. Moreover, the tendency shown for decreased fasting glucose in the TTI treated group might be explained by TNF-α reduction, which decreases insulin resistance [10,12].

Other studies have observed anti-inflammatory effects of trypsin inhibitors [8,19], and bioactive compounds [58] with no alterations in weight. Machado et al. [8] evaluating a kunitz inhibitor from *Erythrina velutina* seeds observed a 41% reduction in TNF-α and no action on IL-6 release. Additionally, this inhibitor reduced monocytes by 52%. Unfortunately, for trypsin inhibitors, to our current knowledge, there are no studies addressing possible signaling pathways that could explain the findings from the present study. Nevertheless, for bioactive compounds, some signaling pathways for TNF-α production have been elucidated [58,59,60]. 

Curcumin, a widely studied polyphenol, in an obese experimental model mimicked the anti-inflammatory effect of a calorie-restricted diet, without changing body weight or adipose tissue weight [58]. Actually, curcumin acts reducing inflammation by inhibiting NF-κB translocation and the consequent transcription of inflammatory cytokines, such as TNF-α [58,59]. An in vitro study has also demonstrated that curcumin binds to IKKβ, inhibiting NF-κB activity [59]. 

Other studies have evaluated the effect of different compounds, such as quercetins and gingerol, which directly act in macrophages, suppressing TNF-α transcription. This is done inhibiting c-Jun *N*-terminal quinase (JNK) phosphorylation, activation, and inhibiting P38 mitogen-activated protein kinase (p38- MAP quinase) activity [58,60]. 

Although many trypsin inhibitors reduce weight gain [15,18,20,21,22], in the present study this was not observed. Hence, the anti-inflammatory effect we observed in this study was independent of weight loss, indicating that there might be unknown signaling pathways for TTI action. One hypothesis to test is that TTI acts in TNF-α pathways, similarly to the bioactive compounds cited above [58,59,60]. Other hypothesis is that TTI regulates TNF-α transcription pathway by inducing PPARγ2, similarly to TZDs [9,10]. Once TZDs are hepatotoxic and TTI so far has no toxicity to animals, this inhibitor might represent a selective less toxic substance to MetS treatment. Therefore, further studies to elucidate transcription pathways of TTI are needed to fully understand its action on decreasing TNF-α shown in this study. In addition, signal transduction promoted by TTI must be investigated. 

Thus, this study offers data that help understanding the effect of satietogenic enzymatic inhibitors. It adds that a natural trypsin inhibitor, besides being satietogenic, might reduce inflammation associated with obese-induced MetS with no apparent toxicity. This contributes to formulation of new therapies for obesity, or even justifies a beneficial effect of an anti-trypsin activity. 

## 5. Conclusions

TTI caused reduction in food intake of a cafeteria diet in animals with obesity-based MetS, corroborating its satietogenic effect seen in eutrophic models. Furthermore, it substantially reduced TNF-α, regardless of weight loss. These effects show that TTI might represent a bioactive protein with potential to be tested as an adjuvant or drug for MetS disorders. 

## Figures and Tables

**Table 1:** Diets used in the experiments.

Ingredients	Cafeteria Diet	Standard Diet
Labina^®^ (g)	45.2	100.0
Condensed milk (mL)	45.2	-
Sugar (g)	9.6	-
Total	100.0	100.0

**Table 2:** Normal values for parameters used for obesity and MetS diagnosis in *Wistar* rats.

Lee Index * (g/cm^3^)	Waist Circumference * (cm)	Fasting Glucose * (mg/dL)	TG * (mg/dL)	HDL * (mg/dL)
0.280 ± 0.0	14.1 ± 0.2	68.0 ± 13.5	44.6 ± 19.6	29.8 ± 4.7

* Data represent mean values from five male eutrophic *Wistar* rats (320–380 g). TG: Triglycerides; HDL-C: high-density lipoprotein.

**Table 3:** Study *Wistar* rats according to measures and biochemical parameters used for metabolic syndrome diagnosis.

Rats	Lee Index (g/cm^3^)	Waist Circumference (cm)	Fasting Glucose (mg/dL)	TG (mg/dL)	HDL-C (mg/dL)
R1	0.303 *	16.0 *	127 *	260 *	43
R2	0.314 *	17.0 *	129 *	192 *	40
R3	0.312 *	16.0 *	158 *	58	35
R4	0.303 *	16.0 *	105 *	45	33
R5	0.313 *	16.5 *	186 *	53	35
R6	0.308 *	16.5 *	167 *	192 *	44
R7	0.302 *	17.0 *	112 *	191 *	33
R8	0.310 *	16.5 *	184 *	147 *	66
R9	0.315 *	16.0 *	151 *	42	22 *
R10	0.306 *	16.0 *	115 *	74 *	28
R11	0.307 *	16.0 *	151 *	106 *	30
R12	0.304 *	16.0 *	123 *	49	25
R13	0.304 *	16.0 *	113 *	42	24 *
R14	0.305 *	16.0 *	99 *	61	29
R15	0.306 *	17.0 *	173 *	44	23 *

* Altered values, considering normal results from Table 1. TG: triglycerides; HDL-C: high-density lipoprotein.

**Table 4:** Biochemical and inflammatory parameters in studied groups after 10 day of experiment.

Parameters	Cafeteria Diet + TTI Mean ± SD	Standard Diet Mean ± SD	Cafeteria Diet Mean ± SD
Fasting glucose (mg/dL)	114.4 ± 34.6	84.0 ± 34.7	203.3 ± 122.0
Total cholesterol (mg/dL)	127.6 ± 62.3	84.6 ± 45.3	102.6 ± 32.8
HDL-C (mg/dL)	31.8 ± 6.4	26.4 ± 8.4	23.4 ± 3.9
LDL-C (mg/dL)	71.2 ± 62.9	45.8 ± 36.4	50.2 ± 31.8
VLDL-C (mg/dL)	24.58 ± 6.9	12.4 ± 3.6 *	29.0 ± 7.1
TG (mg/dL)	123.2 ± 34.6	62.2 ± 18.0 *	145.0 ± 35.3
SGOT (mg/dL)	17.00 ± 1.41	15.80 ± 3.35	19.00 ± 5.34
SGPT (mg/dL)	28.50 ± 17.94	31.40 ± 9.56	43.20 ± 19.20
GGT (mg/dL)	19.25 ± 2.99	22.80 ± 17.11	18.00 ± 5.10
IL-6 (pg/mL)	1.68 ± 0.24	2.25 ± 1.17	1.85 ± 0.13
TNF- α (pg/mL)	undetectable	6.53 ± 0.96 ^#^	5.86 ± 0.43

* Kruskal–Wallis test, followed by Dunn’s post-hoc test, with *p* < 0.05. ^#^ *n* = 4 animals was used, once 1 animal presented undetectable concentrations. Cafeteria diet + TTI (cafeteria diet + 1 mL of gavage TTI at 25 mg/kg); Standard diet (Labina^®^ diet + 1 mL of gavage water); Cafeteria diet (cafeteria diet + 1 mL of gavage water). All groups represent experiments with *n* = 5 animals. HDL-C: high-density lipoprotein; LDL-C: low-density lipoprotein; VLDL-C: very low-density lipoprotein; TG: Triglycerides; SGOT: oxaloacetic transaminase; SGPT: glutamic pyruvic transaminase; GGT: gamma-glutamyl transpeptidase; IL-6: interleukin-6; TNF-α: Tumor necrosis factor-alpha; TTI: Tamarindo seed Trypsin Inhibitor.
